# The Association of ACE Genotypes on Cardiorespiratory Variables Related to Physical Fitness in Healthy Men

**DOI:** 10.1371/journal.pone.0165310

**Published:** 2016-11-18

**Authors:** Salomão Bueno, Leonardo A. Pasqua, Gustavo de Araújo, Adriano Eduardo Lima-Silva, Rômulo Bertuzzi

**Affiliations:** 1 Endurance Sports Research Group, School of Physical Education and Sport, University of São Paulo, São Paulo, Brazil; 2 Sports Science Research Group, Federal University of Alagoas, Maceió, Brazil; 3 Sports Science Research Group, Academic Center of Vitoria, Federal University of Pernambuco, Vitoria de Santo Antao, Brazil; National Cancer Center, JAPAN

## Abstract

Aerobic power (VO_2_max), aerobic capacity (RCP), and running efficiency (RE) are important markers of aerobic fitness. However, the influence of the angiotensin converting enzyme (ACE) polymorphism on these markers has not been investigated in healthy individuals. One hundred and fifty physically active young men (age 25 ± 3 years; height 1.77 ± 0.06 m; body mass 76.6 ± 0.9 kg; VO_2_max 47.7 ± 5.5 ml·kg^-^1·min^-1^) visited the laboratory on two separate occasions, and performed the following tests: a) a maximal incremental treadmill test to determine VO_2_max and RCP, and b) two constant-speed running tests (10 km·h^-1^ and 12 km·h^-1^) to determine RE. The genotype frequency was II = 21%; ID = 52%; and DD = 27%. There was a tendency for higher VO_2_max with the ACE II genotype (p = 0.08) compared to DD and ID genotypes. Magnitude based inferences suggested a *likely beneficial effect* on VO_2_max with the ACE II genotype. There was no association between genotypes for other variable. These findings suggest that individuals with the ACE II genotype have a tendency towards better values in aerobic power, but not with aerobic capacity or running economy.

## Introduction

In the last decade several components of physical fitness have been identified as essential to maintaining healthy life-style [1], including cardiorespiratory endurance and aerobic fitness [2]. Aerobic fitness is related to oxygen transport and utilization [2] and its importance has been associated to cardiovascular system [3]. The most relevant physiological variables related to aerobic fitness include aerobic power, i.e. maximal oxygen uptake (VO_2_max) [2, 3], aerobic capacity, i.e. the respiratory compensation point (RCP) [4] and efficiency of energy use during running, i.e. running economy (RE) [5].

It has been demonstrated that aerobic fitness is a result of the interaction between environmental [6] and genetic factors [7]. In particular, a genetic polymorphism in the angiotensin-converting enzyme (ACE) gene might influence endurance [7]. ACE is a key-enzyme in the renin angiotensin system (RAS), influencing the conversion of angiotensin-I (Ang-I) to angiotensin II (Ang-II), the half-life of bradykinin [7] and the hydro-electrolytic control in the human body [8]. The ACE gene polymorphism is characterized by insertion or deletion of the 287 base pair in intron 16, resulting in three genotypes; II, ID, and DD [8]. This polymorphism is associated with different ACE concentrations [6] and related to distinct prevalence in sports [7]. The ACE II genotype is more prevalent in successful endurance athletes, such as mountaineers and rowers, compared to DD and ID ACE genotypes [9]. Furthermore, superior endurance performance of individuals with the ACE II genotype compared to DD may be related to increased aerobic metabolism function [10, 11, 12]. For instance, studies have demonstrated superior oxygen extraction (a-vO_2_ difference), due to the action of vasodilator factors such as bradykinin [10], higher oxygen supply to the exercising muscles provide by higher capillarity in skeletal muscle [11] and a greater energy supply due to a greater concentration of the enzymes and organelles involved in aerobic metabolism [12]. Taken together, these findings suggest that processes related to oxygen consumption may mediate the relationship between the ACE gene polymorphism and aerobic fitness.

Despite this association between the ACE II genotype and success in endurance sports [7, 9], few studies have investigated the direct relationship between the ACE genotype and aerobic fitness in healthy individuals [10,13]. It is believed that whole-body physiological variables related to oxygen consumption are influenced by the level of training of the individuals [3, 4 5, 6]. In addition, most studies comparing ACE genotypes and aerobic fitness, such as aerobic power, used relatively small cohorts [12, 14, 15, 16] limiting the interpretation of these results. Thus, it appears logical to suggest that the influence of the ACE genotype on VO_2_max, RCP and RE could be more evident in an increased cohort of non-athletic individuals. Furthermore, previous research has primarily employed cycling tests [12, 13, 17]. Cycling is influenced by enhanced muscle tension imposed by the muscle contraction [18], which reduces peripheral blood flow and oxygen extraction [19]. Thus, the mechanical aspects of muscle contraction during cycling might influence oxygen consumption, which may affect the relationship between the ACE polymorphism and aerobic power. Therefore, studies aiming to clarify the association between ACE genotypes and variables associated to aerobic fitness may benefit from employing running tests in non-trained individuals.

The present study aimed to investigate the influence of ACE genotypes on cardiorespiratory variables relating to aerobic fitness during running (VO_2_max, RCP and RE). It was hypothesized that individuals with ACE II genotype would have a greater VO_2_max, RCP and RE in comparison to individuals with ID and DD genotypes.

## Materials and Methods

### Participants

One hundred and fifty healthy young males (age 25.0 ± 3.0 years, body mass 76.6 ± 0.9 kg, height 1.77 ± 0.06 m, and body fat 13.5 ± 3.9%) without muscular disease or cardiovascular pathology were recruited. All participants received verbal explanation about the possible benefits, risks, and discomfort associated with the study and gave written informed consent. Procedures were in accordance with the Helsinki declaration of 1975 and approved by the Local Ethics Committee for Human Studies of the University of São Paulo.

### Experimental Design

The volunteers visited the laboratory on two separate occasions. The first visit consisted of DNA collection, anthropometric measurements and a maximal incremental treadmill test to exhaustion. During the second visit, participants performed two submaximal treadmill tests, separated by six minutes of rest, and completed a short version of the physical activity questionnaire (IPAQ-short version). All tests were performed at the same time of day at a constant room temperature (20–24°C) and 2–3 h following the last meal. The volunteers were asked to refrain from any exhaustive exercise 48 h prior to the tests, and from taking nutritional supplements during the experimental period.

### Anthropometric measurements

Anthropometric measurements were taken according to guidelines described by Norton and Olds [20]. Individual body mass was measured on an electronic scale. Height was measured using a stadiometer. Skinfold thickness was measured at seven sites (chest, axilla, triceps, subscapular, abdominal, suprailiac, and thigh) using a Harpenden caliper (West Sussex, UK). The same evaluator took all measurements. The Jackson and Pollock’s [21] equation was used to estimate body density and Brozek et al. [22] equation was used to estimate body fat.

### Maximal incremental treadmill test

The participants performed a maximal incremental running test on a motor-driven treadmill (TK35, CEFISE, Nova Odessa, Brazil) to determine VO_2_max and RCP. Following a 3-min warm-up at 8 km·h^-1^, speed was increased by 1 km·h^-1^ every minute until exhaustion, which was defined as the individual’s inability to maintain the running pace. Participants received strong verbal encouragement to ensure they continued as long as possible. Gas exchanges were measured breath-by-breath throughout the exercise using a gas analyzer (Cortex Metamax 3B, Cortex Biophysik, Leipzig, Germany) and subsequently averaged over 30-s intervals. Heart rate was monitored during the test using a heart rate transmitter (Polar ^®^Beat, Kemple, Finland) coupled to the gas analyzer. VO_2_max was determined when two or more of the following criteria were met: an increase in VO_2_ of less than 2.1 ml·kg^-1^·min^-1^ between two consecutive stages [23]; a respiratory exchange ratio greater than 1.1 [23]; and a heart rate within 10 bpm of the maximal estimated heart rate of 220-age [23]. Maximal heart rate was defined as the highest value obtained during the test (HR_MAX_). Three independent investigators determined the RCP by a nonlinear increase in VE/VCO_2_, a constant increase in VE/VO_2_, and a first decrease in the expiratory fraction of CO_2_ [4].

### Submaximal test

Participants performed two submaximal runs at 10 km·h^-1^ and 12 km·h^-1^ for 6 min interspersed by a 6-min interval. The device used to measure the VO_2_ was the same used for the incremental test. The RE was calculated as the mean VO_2_ over the last 30-s of each exercise speed [5].

### DNA extraction

The cells for DNA extraction were obtained via mouthwash. Cells were submitted to an overnight digestion with proteinase K. Nucleotides were separated from the cellular debris by density gradient centrifugation using chloroform. Genomic DNA was precipitated with isopropyl alcohol, isolated by centrifugation and resuspended in TE buffer. DNA quantification was performed using a spectrophotometer (NanoDrop, ND 2000, USA), and the concentration was adjusted to 1 μg.μL^-1^ for subsequent storage in -20°C.

ACE gene polymorphisms were determined in PCR-real time (Polymerase Chain Reaction) by 3-primer PCR described previously (ACE-1: 5'-CATCCTTTCTCCCATTTCTC-3'; ACE-2: 5'-TGGGATTACAGGCGTGATACAG-3' and ACE-3: 5'-ATTCAGAGCTGGAATAAAATT-3'). The Platinum SYBR-green reagent (Life Technologies^®^) was utilized for amplification of the insertion or deletion genotypes. The SYBER-green fluoresces is quantified from the dissociation curve, with allele I showing dissociation at approximately 73°C, allele D at approximately 76°C and subjects with two alleles submit two dissociation temperatures (73°C and 76°C).

### Statistical Analysis

Participants were separated according to ACE genotypes (II, ID or DD). Hardy-Weinberg equilibrium was tested using the *chi-square test*. Data normality was assessed via the Kolmogorov-Smirnov test. Because all variables showed a normal distribution, data are reported as means and standard deviations (±SD) and parametric tests applied. One-way analysis of variance (ANOVA) was used to compare the effects of the ACE genotype on VO_2_max, RCP, and RE. The significance level was set at α ≤ 0.05 and all statistical analyses were performed using SPSS (version 13.0; Chicago, IL).

In addition, the smallest worthwhile effects were calculated for each physiological variable between pairs of genotype (II *vs* DD, II *vs* ID, DD *vs* ID) to determine the likelihood that the effect was substantially beneficial, trivial or detrimental [24]. Quantitative chances of beneficial effects were assessed qualitatively as follows: <1% almost certainly not, 1–5% very unlikely, 5–25% unlikely, 25–75% possible, 75–95% likely, 95–99 very likely, and >99% almost certain. Furthermore, if the chance of having beneficial and detrimental effect were both > 5%, the true difference was assessed as unclear [24].

## Results

The genotype frequencies attained using the Hardy-Weinberg Equilibrium were: II = 21%; ID = 52%; DD = 27%. The frequency of the ACE genotype was similar to that reported previously in a Caucasian population[21]. Participants’ characteristics were similar between groups (p > 0.05) (Table 1). Level of physical activity was classified as moderately active in accordance with mean values of the IPAQ-SV outcomes (Table 1).

**Table 1:** Anthropometric characteristics, physical activity level, and physiological variables of the participants (n = 150).

	DD (n = 41)	ID (n = 77)	II (n = 32)n = 32	*p Value* value
**Age (years)**	24 ± 4	25 ± 3	26 ± 3	0.12
**Mass (kg)**	75.8 ± 8.9	78.1 ± 9.7	73.9 ± 8.3	0.07
**Heights (cm)**	177.4 ± 5.1	177.2 ± 5.9	175.2 ± 4.8	0.16
**Body fat (%)**	13.1 ± 4.3	14 ± 3.8	13 ± 3.6	0.27
**IPAQ-SV (score)**	1320 ± 120	1298 ± 134	1302 ± 153	0.72

IPAQ-SV: international physical activity questionnaire—short version

**Fig 1:**
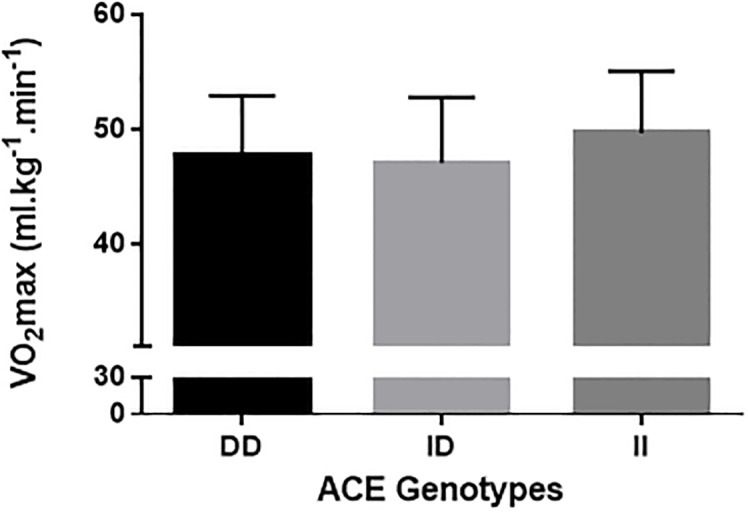
VO_2_max.

**Fig 2:**
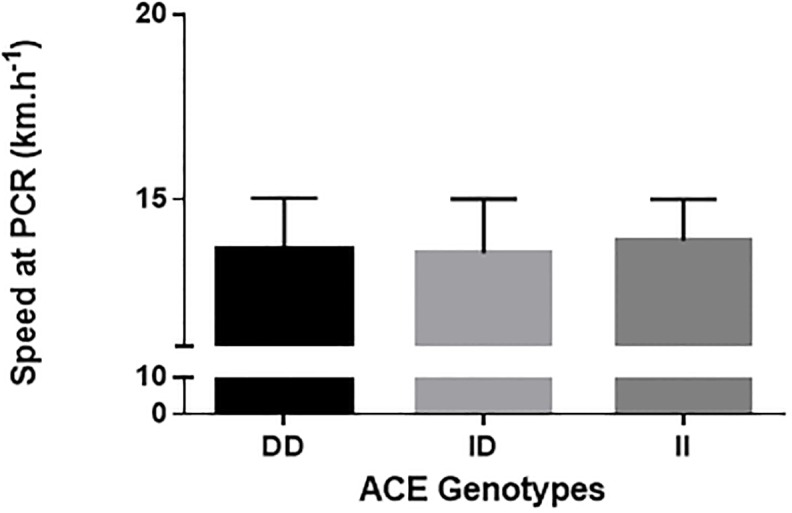
RCP.

**Fig 3:**
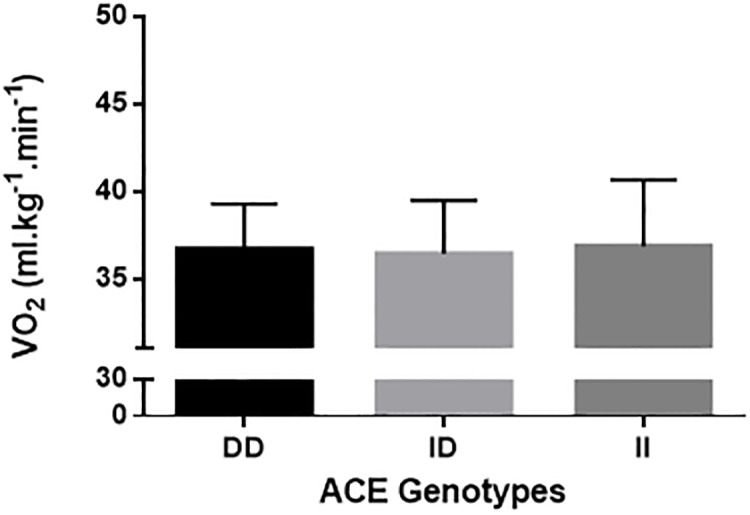
RE at 10 km.h^-1^.

**Fig 4:**
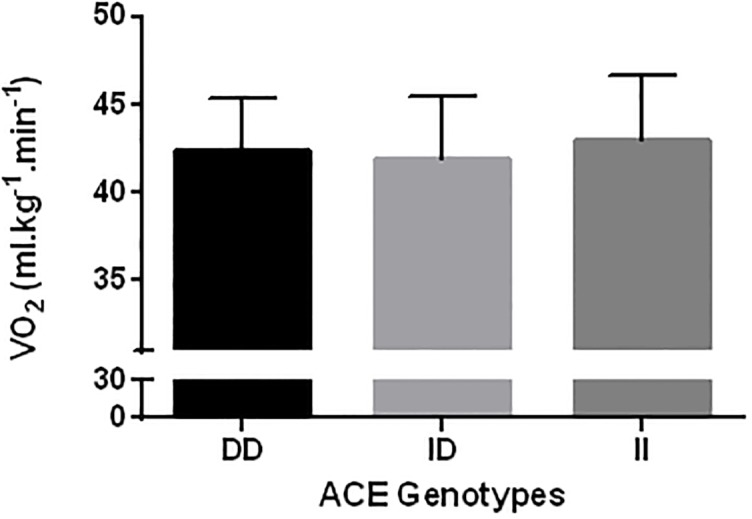
12 km.h^-1^. Comparison between ACE DD, ID and II genotypes. VO_2_max: maximal oxygen uptake; RCP: running speed of respiratory compensation threshold; RE_10km.h_^-1^: running economy at 10 km.h^-1^; RE_12km.h_^-1^: running economy at 12 km.h^-1^.

Figures shows the comparisons of VO_2_max (Fig 1), RCP (Fig 2), RE_10km.h_^-1^(Fig 3), and RE_12km.h_^-1^ (Fig 4) for each ACE genotype. There was a trend towards higher VO_2_max values in the ACE II genotype compared to the DD and ID genotypes (p = 0.086). There were no statistical differences between genotypes for RCP, RE_10km.h_^-1^, and RE_12km.h_^-1^ (all p > 0.05).

Table 2 shows the chances of a beneficial/trivial/ detrimental effect for each physiological marker between ACE genotypes. Comparison of VO_2_max between ACE II and DD genotypes showed an 88% likelihood of being beneficial, 9% trivial and 3% harmful for ACE II. These data demonstrate a likely beneficial effect of the ACE II genotype on VO_2_max (Table 2). For all other comparisons, unclear effects were obtained.

**Table 2:** The chance of likelihood effect for each cardiorespiratory variables.

	The chance of genotype being beneficial (%)	The chance of genotype being trivial (%)	The chance of genotype being detrimental (%)	Qualitative descriptor
**VO_2_max**				
**II vs DD**	88	9	3	Likely-Beneficial
**II vs ID**	52	32	16	Possible-Unclear
**ID vs DD**	16	32	52	Possibly-Unclear
**RCP**				
**II vs DD**	15	81	1	Likely-Trivial
**II vs ID**	10	89	1	Likely-Trivial
**ID vs DD**	0	72	28	Possible-Trivial
**RE_10km.h_^-1^**				
**II vs DD**	26	47	27	Possible-Trivial
**II vs ID**	28	62	10	Possible-Trivial
**ID vs DD**	0	72	28	Possible-Trivial
**RE_12km.h_^-1^**				
**II vs DD**	46	43	11	Possible -Unclear
**II vs ID**	34	48	18	Possible-Trivial
**ID vs DD**	09	51	40	Possible-Trivial

## Discussion

Previous research has suggested that the ACE polymorphism might be a relevant genetic factor for aerobic fitness in humans [7, 17], specifically that the ACE II genotype might have beneficial effects on oxygen consumption [10, 13]. The major finding of this study was that individuals with the ACE II genotype showed an 88% likelihood of having a higher VO_2_max compared to the DD genotype.

It has been suggested that genetic factors may influence up to 50% of the variability of the individual training-induced increases in aerobic power [25, 26]. In particular, the ACE polymorphism has been considered as one of the most relevant [7]. It has been demonstrated that lower concentrations of ACE, common in the ACE II genotype, influences the half-life and concentration of bradykinin [12]. Individuals carrying these genotypes, due to the higher concentration of bradykinin, have a better vessel permeability [10] and oxygen supply [25]. Therefore, it is believed that increased oxygen availability to the skeletal muscle during exercise contributes to an increased VO_2_max [10]. Support for this hypothesis was shown by Hagberg et al. [10], who showed that postmenopausal women with the ACE II genotype had greater VO_2_max values than their DD or ID genotype counterparts. These authors also showed that the greater VO_2_max values were associated with higher arteriovenous oxygen differences in II genotype compared to the other two ACE genotypes. Similarly, in the present study, individuals with the ACE II genotype tended to have a greater aerobic power compared to individuals with ID and DD genotypes. These differences were shown to be beneficial as suggested by magnitude based inferences (88% vs. DD).

Controversially, there are some findings indicating that the ACE DD genotype influences VO_2_max in healthy adults [12, 21], while others have not demonstrated any association between ACE genotypes and VO_2_max [16, 27]. For instance, Vaughan et al. [12] and Zhao et al. [14] showed that individuals with ACE DD genotype have a greater VO_2_max than those with the ACE II genotype. These results occur primarily because the ACE DD genotype is related to left ventricle hypertrophy (LVH) [14] and LVH has been shown to be positively correlated tocardiac output leading to possible benefits in endurance performance [6]. Nevertheless, it is well known that increased LVH does not represent an increase in VO_2_max More studies are necessary to confirm this association with ACE genotype [7].

It is important to emphasize that Rankinen et al. [16], Vaughan et al. [12] and Day et al. [27] determined VO_2_max on a cycle ergometer, which requires increased concentric muscle contraction [18]. It has been shown that critical occlusion of blood flow occurs at approximately 45–60% of maximal voluntary contraction [28]. Thus, a higher percentage of maximal voluntary contraction is required during cycling as compared to running [19], which could mechanically reduce peripheral blood flow and oxygen availability. Therefore, while the exact mechanisms that underlie the differences between the current and previous studies have not been completely elucidated, it is plausible to assume that the ACE II genotype may have an important role in oxygen delivery to the exercising muscle in physically active individuals, which results in elevated values of aerobic power.

Studies have shown that the ACE polymorphism influences metabolic [11], and peripheral muscle adaptation [29]; individuals with the ACE II genotype present differences in muscle fiber distribution [11] and concentration of aerobic-associated enzymes [12]. Zang et al. [29] compared muscle fiber composition in individuals with the II, ID and DD ACE polymorphism and showed that individuals with the II genotype showed a greater quantity of type IA fibers than ID ad DD. Similarly, Vaughan et al. [12] found that individuals with the II genotype show a modulation in capillary supply lines, resulting in beneficial adjusts to lipid metabolism. Therefore, individuals with the ACE II genotype seem to be more susceptible to peripheral adaptations compared to DD or ID genotypes [13]. It could thus be expected that these peripheral differences would influence submaximal physiological variables such as energy cost and aerobic capacity [17]. Williams et al. [13] found that individuals with the ACE II genotype had a higher delta efficiency, which was estimated as the percentage ratio of the change in work performed per minute to the change in energy expended per minute. This indicates that individuals with the ACE II genotype are energetically more economical than individuals with other ACE genotypes. However, the results of the present study showed no differences in variables related to aerobic capacity or running economy during running (i.e. RCP, RE_10km/h_, and RE_12km/h_) between the three ACE genotypes. These findings are in agreement with the results of Gomez-Gallego et al. [17] and Orysiak et al. [30], who showed no differences between ACE genotypes for power output at the ventilatory threshold and RCP in athletes. Taken together, the findings of the current study suggest that, in the present cohort, the ACE genotypes did not influence the submaximal variables important to aerobic fitness that were measured.

## Conclusion

In conclusion, the results of the current study showed that individuals with the ACE II genotype tended to have a greater VO_2_max compared to individuals with the ACE DD genotype. Despite this, the ACE genotypes did not appear to have any influence on submaximal variables related to physical fitness. Thus, further studies are required to clarify the influence of the ACE polymorphism in submaximal variables related to aerobic fitness.

## Supporting Information

S1 Supporting Information (XLSX)
